# Optimizing energy efficiency in induction skull melting process: investigating the crucial impact of melting system structure

**DOI:** 10.1038/s41598-024-56966-7

**Published:** 2024-03-15

**Authors:** Chaojun Zhang, Lunyong Zhang, Fuyang Cao, Zhishuai Jin, Guanyu Cao, Hongxian Shen, Yongjiang Huang, Jianfei Sun

**Affiliations:** 1 National Key Laboratory of Precision Hot Processing of Metals, Harbin Institute of Technology, Harbin, 150001 China; 2 School of Materials Science and Engineering, Harbin Institute of Technology, Harbin, 150001 China

**Keywords:** Induction skull melting technology, Structure optimization, Electromagnetic intensity and uniformity, Charge energy utilization, Mechanical engineering, Techniques and instrumentation

## Abstract

Induction skull melting (ISM) technology could melt metals with avoiding contamination from crucible. A long-standing problem of ISM is that the low charge energy utilization and inhomogeneous fields have obstructed its application in many critical metal materials and manufacturing processes. The present work investigated the problem through the structure optimization strategy and established a numerical electromagnetic-field model to evaluate components’ eddy current loss. Based on the model, the effect of crucible and inductor structure on charge energy utilization, etc. was studied. Furtherly, the charge energy utilization was increased from 27.1 to 45.89% by adjusting the system structure. Moreover, structure modifications are proposed for enhancing electromagnetic intensity and uniformity, charge soft contact and uniform heating. The work constructed a basis for framing new solutions to the problem through ISM device structure optimization.

## Introduction

In order to improve service performance and energy-saving, the application of lightweight materials is urgent in the aerospace and automobile industry ^1,2^. Furthermore, induction skull melting (ISM) is widely used for processing high melting point and reactive metals such as Ti, Zr, Mo, etc. ^3,4^ to produce excellent performance castings working in critical fields such as aerospace vehicles ^5–7^. The unique feature of ISM technology is a solid skull layer between melt and crucible, thus, avoiding molten melt contamination ^8^. Meanwhile, the molten melt is stirred by electromagnetic (EM) force, which caused a better composition homogeneity ^9^. However, the well-known limitations of ISM are remained up to now, low charge energy utilization and inhomogeneous fields above the charge have a negative impact on the energy utilization and charge heating process. Therefore, elimination or reduction of the limitations by optimizing melting system structure is desired.

Due to high temperature and vacuum conditions, modern metallurgical technology mostly uses numerical simulation methods to optimize the parameters of such furnaces ^10–12^. For the past years, researchers have mostly focused on aspects such as EM distribution ^13^, heat and mass transfer ^14–18^, melt free surface phenomenon ^19,20^, turbulence ^21^, superheat ^22–24^, and evaporation process ^25^, etc. with different numerical methods including finite element method (FEM) ^26,27^, finite volume method (FVM) ^14^, large eddy simulations (LES) ^28^ and meshless methods ^29,30^ and so on. However, the effect of cold crucible and inductor structures on magnetic potential and charge energy utilization was seldom reported. It is infinitely detrimental to the efficiency of EM energy utilization in the induction heating process. It has been shown that reducing crucible mass and adding magnetic shunts could reduce energy loss ^31^. Also, a thinner crucible leads to a higher charge efficiency ^32,33^. These studies set down the foundations for crucible and induction coil structural optimization.

The present work established a numerical model to evaluate the effect of crucible and induction coil structure on magnetic intensity and charge energy utilization. Also, methods to improve the charge energy utilization, magnetic strength and uniformity are further proposed to gain the heating process.

## Numerical calculations

### Mathematical model

The energy utilization calculation with ISM melting was achieved by volume fraction calculations of each component eddy current loss, as shown in Eq. (1). Therefore, comparison of the charge energy utilization in different crucible and coil structures could be done by modelling the EM field.

1 $$ Q_{v} = \int_{V} {\left( {J_{e} \cdot E_{e} } \right)dV} $$

where *Q*_*v*_ is the eddy current loss (W), *J*_*e*_ and *E*_*e*_ are the current density (A/m^3^) and the electric intensity (V/m^3^) in unit volume.

Vector potential was introduced to calculate the electromagnetic field based on Maxwell equations.

2 $$ {\varvec{B}} = \nabla \times {\varvec{A}} $$

3 $$ {\varvec{E}} = - j\omega {\varvec{A}} $$

4 $$ \nabla \times \left( {\frac{1}{\mu }\nabla \times {\varvec{A}}} \right) = {\varvec{J}} $$

5 $$ {\varvec{J}} = \sigma {\varvec{E}} + j\omega {\varvec{E}} + {\varvec{J}}_{s} $$

where ***B*** is the magnetic induction (T), ***A*** is the magnetic vector potential (V s m^−1^), ***E*** is the electric field intensity (V m^−1^), ***J*** is the current density (A m^−2^), ***J***_***s***_ is the current density source (A m^−2^), *ω* is the frequency (Hz), *μ* is magnetic permeability (H m^−1^), *σ* is electrical conductivity (S m^−1^).

The EM force was given in the following equation.

6 $$ {\varvec{F}}_{{{\varvec{EM}}}} = \frac{1}{2}{\text{Re}} \left( {{\varvec{J}}_{{\varvec{c}}} \times {\varvec{B}}_{{\varvec{c}}}^{*} } \right) $$

where ***F***_***EM***_ is the force density averaged over time (N m^−3^), ***B*** is the magnetic induction (T), ***J*** is the current density (A m^−2^), B^*^ is the complex conjugate, and *c* represents the charge.

### Geometry and simplifications

**Figure 1:**
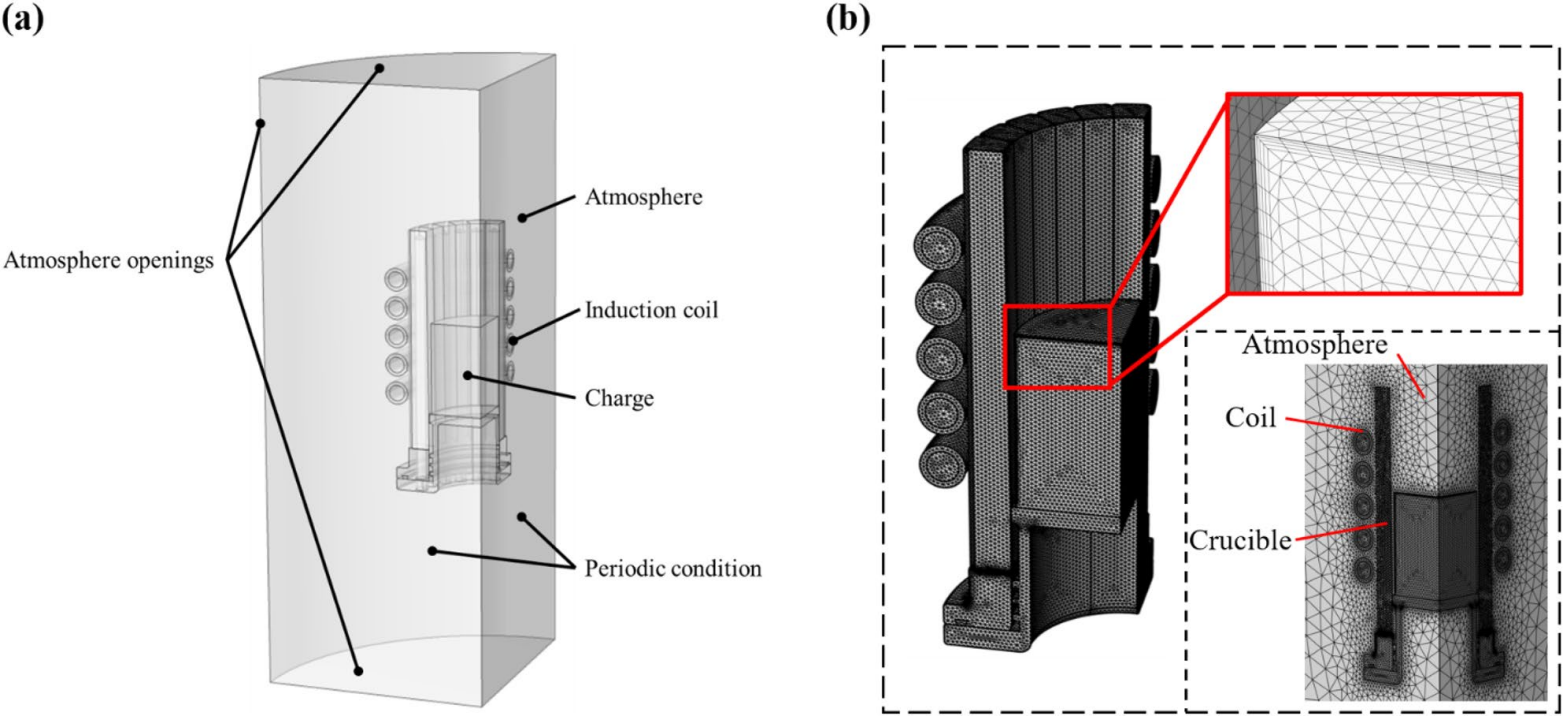
(**a**) The simplification model and (**b**) grid division.

A cold crucible with variable structures was studied in this study for optimization. Due to the slits of the copper crucible, it cannot be simplified to a 2-D model in numerical calculations. Thanks to the centrosymmetric structure of crucible, only a quarter of the geometry model was selected for calculations. In EM field calculations, the research domain should contain all elements including atmosphere (Argon protector), induction coil, crucible and charge. The initial model for EM calculations is shown in Fig. 1a. And the specific dimensional details of the initial model are shown in Table 1. Specific dimensional information can be found in previous publication ^34^.

**Table 1:** Main parts parameters of the initial model.

Main parts	Parameters	Value (mm)
Cold crucible	Inner cavity diameter	97
	Outer diameter	132
	Inner cavity height	160
	Crucible height	220
	Slitting width	0.4
	Slitting depth	172
Copper coil (5 turns)	Height	135
	Outer diameter	20
	Cooling channel diameter	14
	Turn spacing	5

Mesh discretization is vital in numerical simulations, which determines the calculation accuracy. In EM field calculations, the skin layer depth must be taken into account, which is calculated as follows.

7 $$ \lambda = \sqrt {\frac{2}{\omega \mu \sigma }} $$

where *λ* is the depth of the skin layer (m), *ω* is the frequency (Hz), *μ* is magnetic permeability (H m^−1^), *σ* is electrical conductivity (S m^−1^).

In the calculations, the skin layer depth usually contains at least two layers of mesh. In our previous study ^22^, the mesh division using a minimum size of 0.5 mm was able to pass the independence verification. So, we inherited the previous meshing method and optimized the skin layer meshing according to the calculation results of Eq. (7). Furthermore, the infinite domain condition was set at the open boundaries. The mesh was divided as shown in Fig. 1b.

### Boundary conditions and material properties

For atmosphere openings and periodicity conditions in Fig. 1a, the zero magnetic vector and periodicity boundary conditions were introduced, as shown below.

8 $$ {\varvec{n}} \times {\varvec{A}} = 0 $$

9 $$ {\varvec{A}}_{{{\varvec{b1}}}} = {\varvec{A}}_{{{\varvec{b2}}}} $$

where ***n*** is the normal vector, *b*_1_ and *b*_2_ represent different periodicity boundaries.

For material properties, the crucible and coil were made of copper with electrical conductivity of 5.998 × 10^7^ S/m, and density as 8960 kg/m^3^. And the parameter was measured as 2.22 × 10^5^ S/m for the charge (Vit1 metal) by the physical properties measurement system, density as 6250 kg/m^3^.

## Measurement

**Figure 2:**
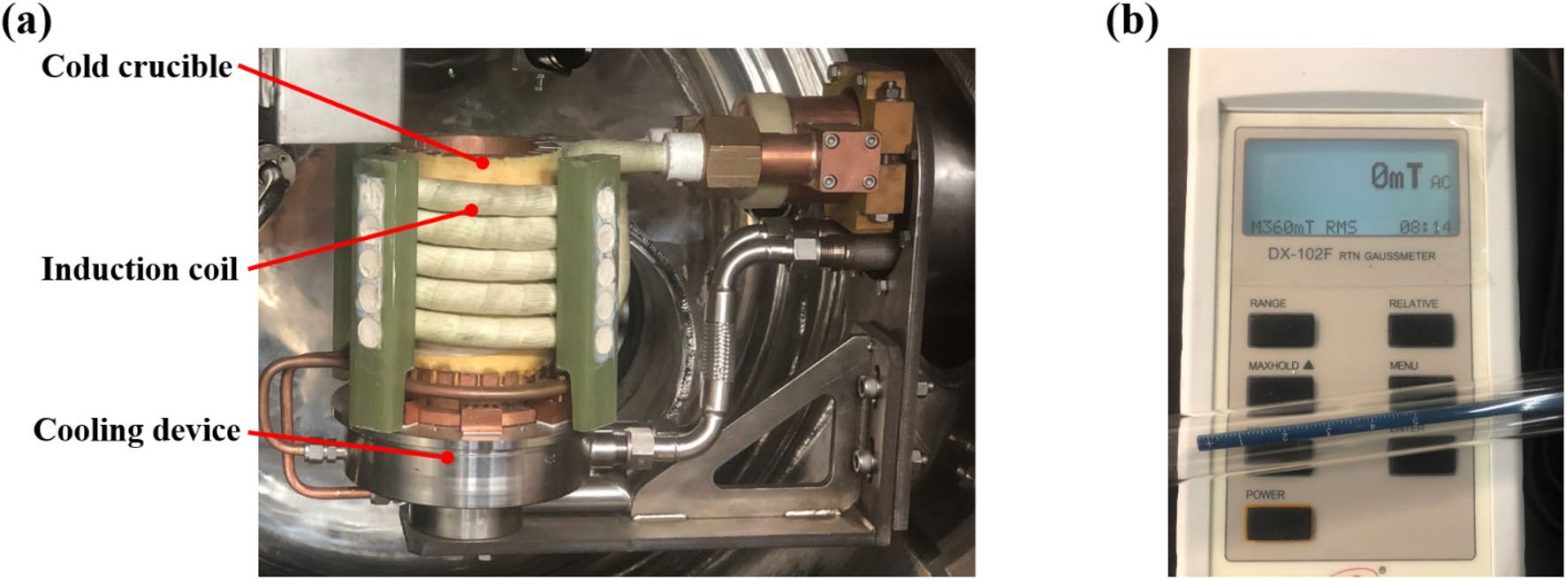
(**a**) ISM furnace with a cold crucible, (**b**) DX-120F Gauss meter.

The experiments were carried out on an ISM furnace equipped with cold crucible, induction coil and cooling device, as shown in Fig. 2a. The induction coil has the capability to deliver a maximum induction power of 180 kW and frequency of 10 kHz, enabling the heating of 2 kg alloys (in terms of titanium). The cooling device ensures complete cooling of the coil and crucible to prevent burn out, as well as facilitating the creation of a skull, resulting in a nearly contamination-free melting of the charge.

The magnetic intensity accuracy measurements were carried out by a Gauss meter (DX-120F), which has a maximum error of 0.16% and suitable for 360 mT AC magnetic field with 10 kHz sampling bandwidth, as shown in Fig. 2b. Also, the Gauss meter has been corrected in zero magnetic environment. We carried out measurements at low induction power in empty crucible for safety. The position was chosen to be located on the central axis of the crucible.

## Results and discussion

All of the simulations were performed on one node of an Intel Xeon Silver 4208 CPU @ 2.10 GHz with 2 T of RAM installed, containing 64 cores. The numerical model was calculated by using *COMSOL Multiphysics*, which has powerful post-processing capabilities and is based on the FEM method for calculations. Under varying induction power levels, the magnetic induction intensity exhibited a C-shaped distribution trend at the crucible central axis and simulation results have passed the consistency test, as reported in previous article ^34^.

### Crucible section

**Figure 3:**
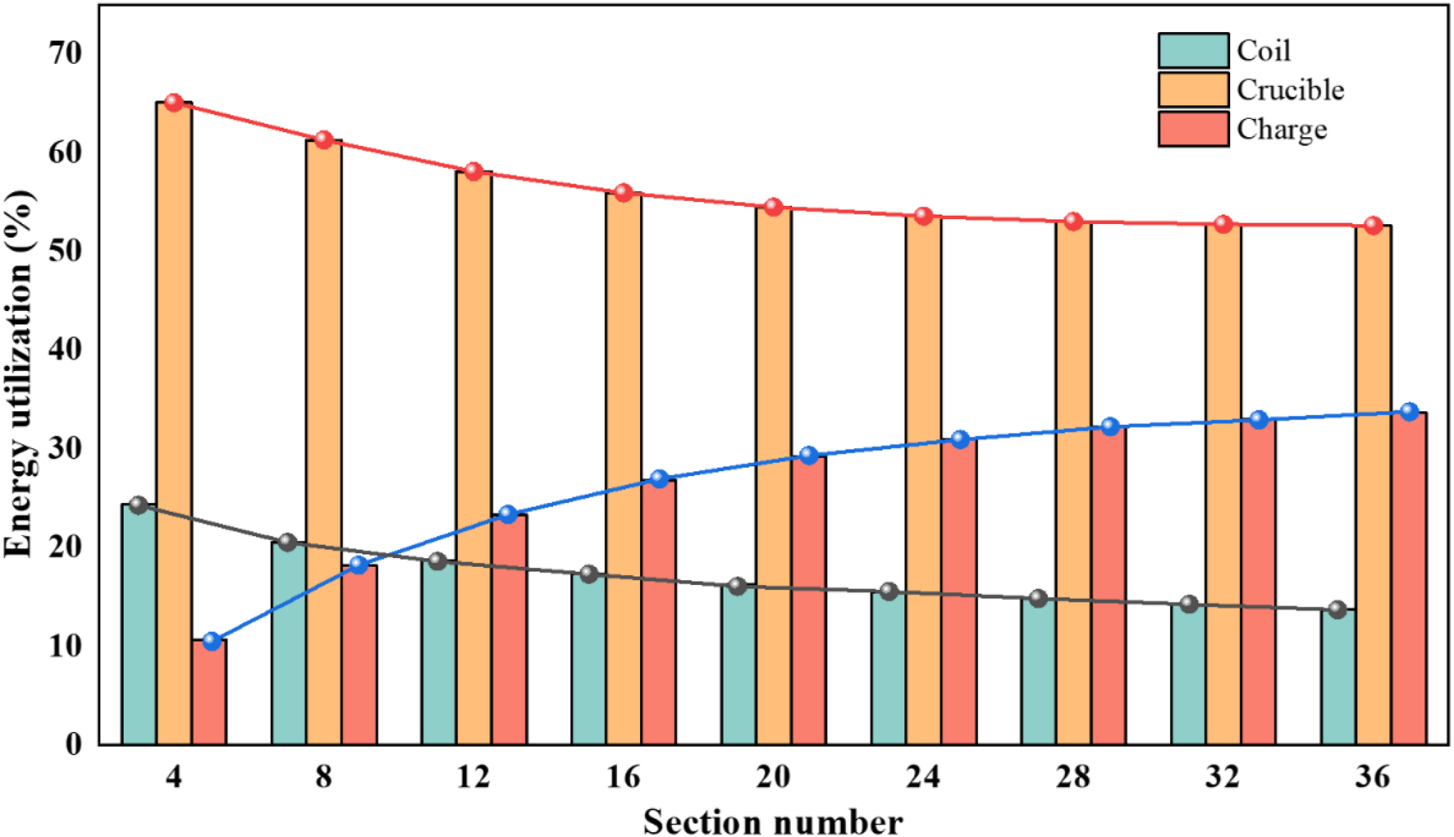
Variation of energy utilization with different crucible section number.

The number of crucible sections (slits) directly affects the penetration of magnetic flux. We explored the component parts' energy utilization, including crucible, induction coil and charge at different crucible section number with 40 kW induction power and 5 kHz frequency, as shown in Fig. 3. As the section number increases, the energy utilization of crucible and coil tend to decrease and the energy utilization of charge tends to increase. It can be inferred that as the section number rises, more magnetic potential is applied to the charge. However, the increase of charge energy utilization is no longer significant when the number is too large since the magnetic potential inside the crucible becomes saturated. It is due to the fact that the magnetic potential inside the crucible is saturated, which is essentially the same as the potential that can enter inside the crucible from the inductor magnetic excitation in this condition. The remaining energy acts on the crucible, the coil and other power-consuming devices.

**Figure 4:**
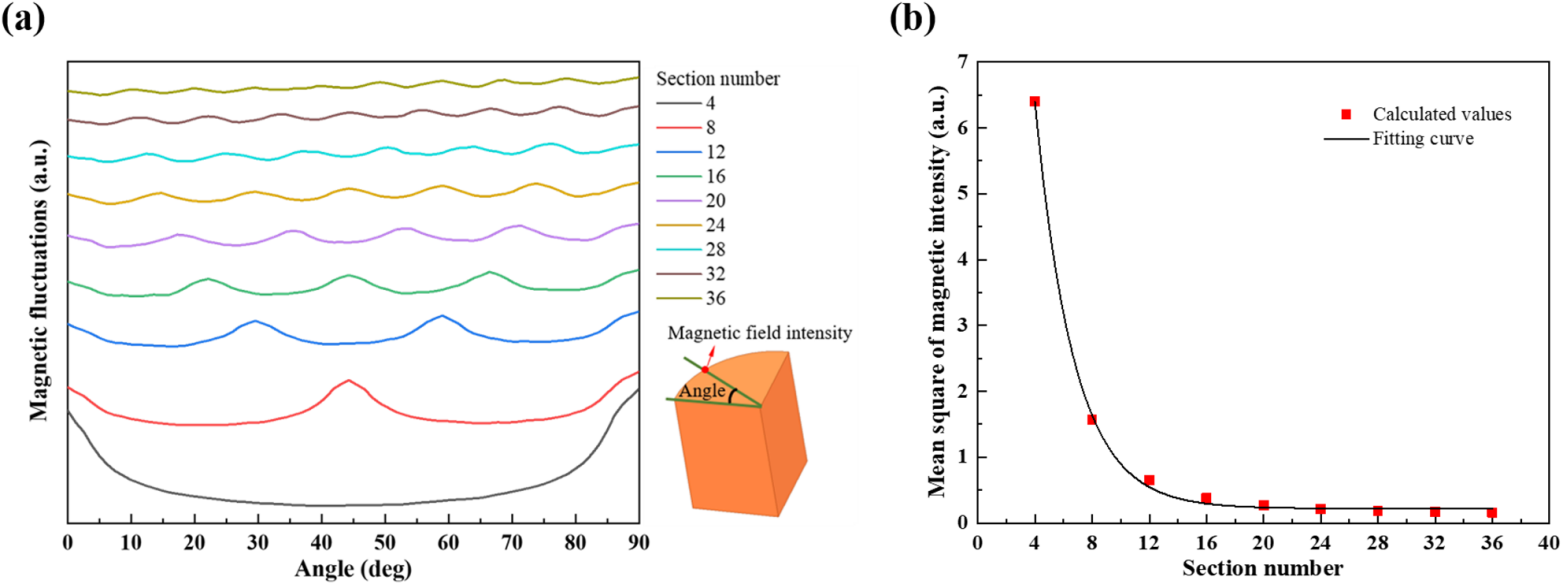
(**a**) Fluctuation of the magnetic intensity in the circumferential direction of the charge and (**b**) the variation of the mean squared deviation with the crucible section number.

The magnetic intensity analysis of the charge top surface shows that the magnetic fluctuates bitterly in the circumferential direction, and there exist magnetic intensity peaks at the crucible slits, as shown in Fig. 4a. The mean squared deviation was introduced to describe the uniformity of the magnetic intensity in this position. The calculation formula is as follows.

10 $$ \delta = \sqrt {\frac{{\sum\nolimits_{i = 1}^{n} {\left( {x_{i} - \overline{x}} \right)^{2} } }}{n}} $$

where *δ* is the mean squared deviation, *x*_*i*_ is the sample value, $\overline{x}$ is the sample average value and *n* is the sample number.

The mean squared deviation values of the magnetic intensity in the circumferential direction and the fitting curve are shown in Fig. 4b. As the crucible section number increases from 4 to 12, the mean squared deviation value decreases significantly; while from 16 to 36 section number, the value decreases slowly, suggesting an enhanced magnetic potential in the crucible with increasing the section number and has a positive effect on increasing the charge magnetic uniformity. However, when the section number is too large, the magnetic potential inside the crucible gradually approaches saturation. After that, it is no longer possible to obviously improve the magnetic permeability of the crucible by increasing the number, as shown in Table 2.

**Table 2:** The maximum magnetic intensity above the chare with different crucible section number.

Section number	4	8	12	16	20	24	28	32	36
Magnetic intensity (mT)	36.35	41.92	46.39	49.60	51.94	53.75	55.05	56.17	56.32

### Slits and curved shape in the crucible bottom

**Figure 5:**
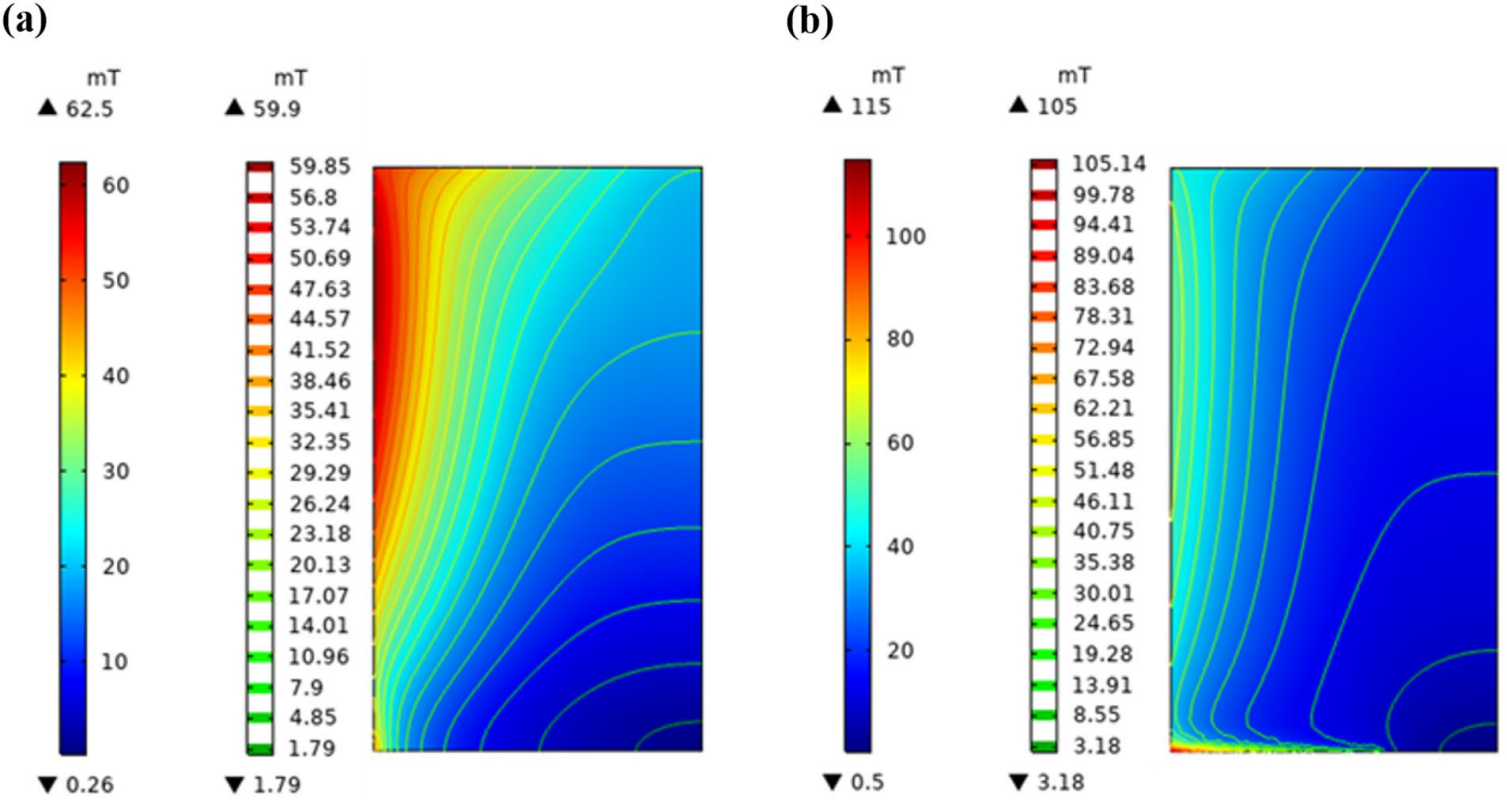
The charge magnetic intensity (**a**) without crucible bottom slits or (**b**) with slits.

When there exist slits in the crucible bottom, the magnetic flux is able to act on the charge through this location. The magnetic intensity above the charge with bottom slits is more evenly distributed in the vertical direction than without, and it results in a higher magnetic intensity at the bottom of the charge. In this case, the charge energy utilization is increased by 1.2%. The comparison of the charge magnetic intensity is shown in Fig. 5 in two cases.

**Figure 6:**
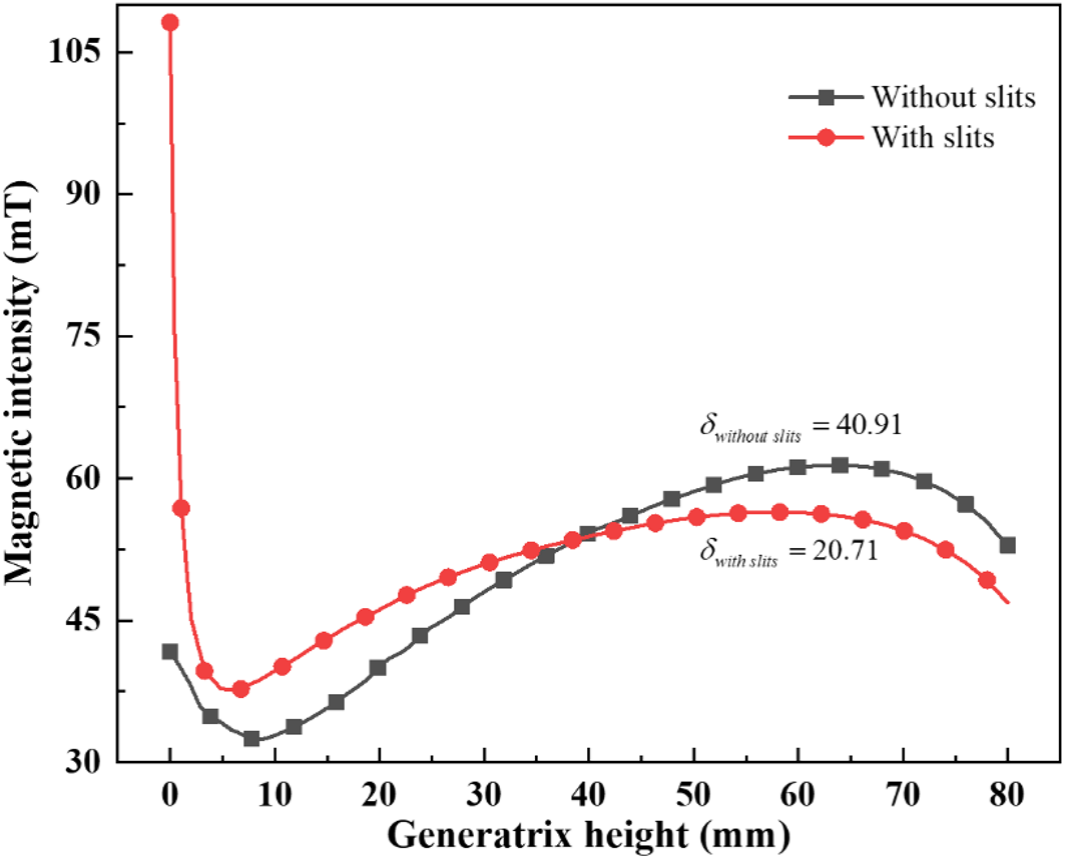
Variation of the magnetic intensity with generatrix height of the charge.

As can be observed from the contours in the diagram, the magnetic flux is more concentrated at the charge bottom when there exist slits in the crucible bottom. The variation of magnetic intensity on the outside of the charge is shown in Fig. 6. The magnetic intensity is more uniform above the charge when there exist slits. At the same time, the introduction of new skin layer at the charge bottom allows the formation of a current density convergence zone at this position, which has an inductive heating effect. Therefore, a new heat transfer trend appears in this way, and the skull thickness could be further reduced during the melting process.

**Figure 7:**
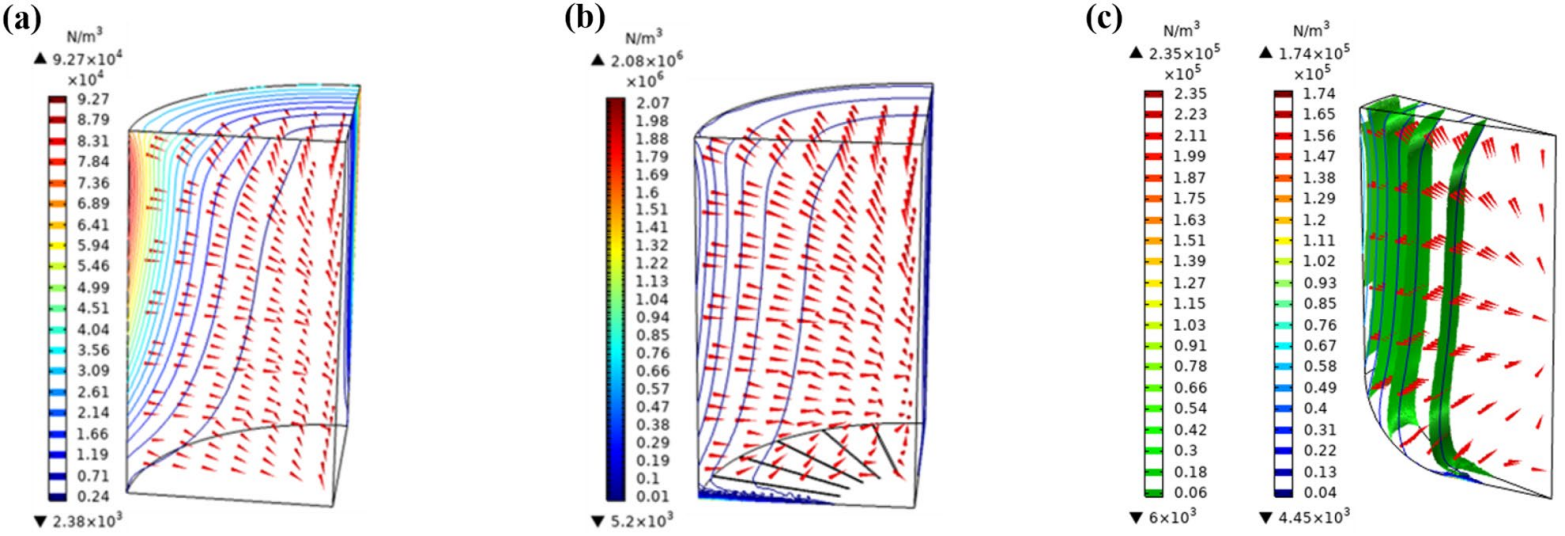
(**a**) Electromagnetic force above the charge with slits in the crucible bottom or (**b**) without, (**c**) electromagnetic force above the charge with crucible bottom rounding treatment.

As shown in Fig. 7a,b, the lifting force in charge bottom is enhanced by introducing slits, which then increases the micro-air gap between charge and crucible and reduces the skull thickness. This was beneficial in improving superheat ^22^. Moreover, it was shown that a curved crucible bottom could induce EM force along the normal direction of curved surface and so causes further increased force on the charge, as shown in Fig. 7c. However, such a design of curved bottom crucible will increase the crucible mass and increase its eddy current loss ^35^.

### Thin-wall crucible with wide slits

**Figure 8:**
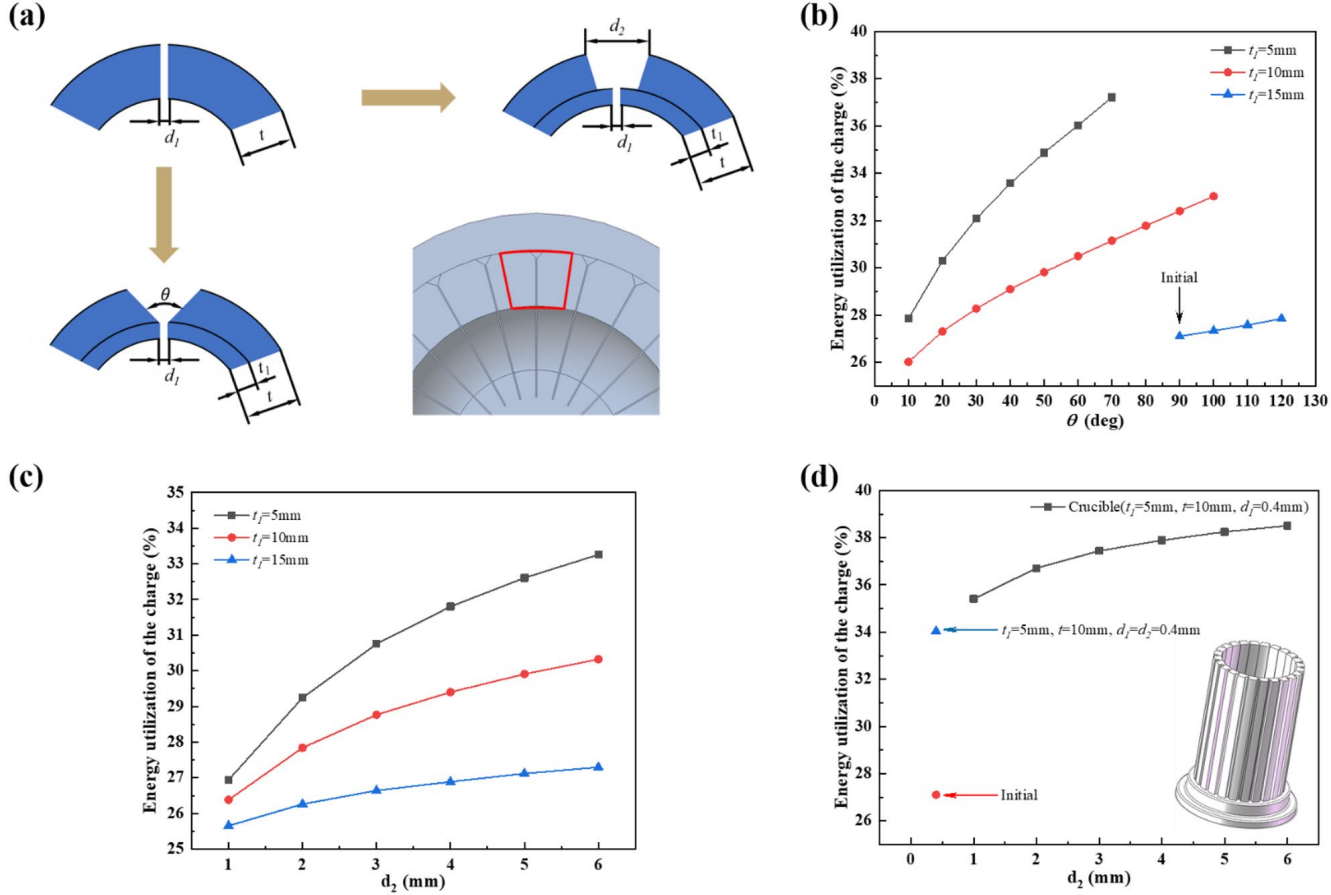
(**a**) The schematic diagram of two type crucibles, the charge energy utilization with (**b**) *t*_1_ − *θ* type and (**c**) *t*_1_ − *d*_2_ type crucible, (**d**) *d*_2_ scanning results with *t*_1_ − *d*_2_ type crucible.

The slits of each copper sector vertical adjacent surfaces consume most of eddy current losses in each sector, resulting in a high crucible temperature ^36,37^. However, excessively wide slits tend to get internal melt to leak out or produce over-burning of the filling material between the slits. Furthermore, the disadvantage of the narrow slits is the low charge energy utilization and the high-power supply required for the same conditions. Previous researches have also shown that the crucible mass is also a fatal factor in the electrical efficiency and should be reduced as much as possible when optimizing the crucible structure ^38^. Therefore, the crucible slit size is increased to reduce the crucible mass. Meanwhile, the wide slit structure also serves to converge the magnetic flux in this region and increases the charge magnetic intensity. So, two configurations of the slit were proposed in this subsection, *t*_1_ − *θ* slit type (*t*_1_: crucible wall thickness for the first slit width; *θ*: slit angle) and *t*_1_ − *d*_2_ type (*d*_2_: second slit width), respectively, with the internal slit remaining narrow (0.4 mm), as shown in Fig. 8a. The initial crucible size as *t* = 17.5 mm (*t*: total wall thickness of crucible) and the variation of the charge energy utilization with *t*_1_ and *θ* is shown in Fig. 8b. For the initial size crucible (*t*_1_ ≈ 15 mm, *t* = 17.5 mm, *d*_1_ = 0.4 mm (*d*_1_: first slit width), *θ* = 90°), the charge energy utilization is 27.1%. As *t*_1_ decreases and *θ* increases, the energy utilization of the charge increases. The smaller the *t*_1_, the more significant the increase in the charge energy utilization. Therefore, the reduction in crucible mass could improve the charge energy utilization. Analysis of *t*_1_ − *d*_2_ type crucible shows that the trend in charge energy utilization is approximately the same as in the *t*_1_ − *θ* type, as shown in Fig. 8c. In view of the mechanical processing and the arrangement of the cooling channels, the *t*_1_ − *θ* type will tend to create sharp corners on the crucible edge when *t*_1_ decreases and *θ* increases, and the cooling channels have to be double-piped, resulting in a poor cooling effect of the crucible. Therefore, the *t*_1_ − *d*_2_ type was used for crucible optimization in subsequent studies. As already mentioned, the reduction of the crucible mass could increase the charge energy efficiency. So, the crucible was modified to a thin-wall construction. Fixing several parameters (*t*_1_ = 5 mm, *t* = 10 mm, *d*_1_ = 0.4 mm) and exploring the effect of changes in *d*_2_ on the energy utilization of the charge to determine the optimum value of *d*_2_. When the crucible structure parameters as *t*_1_ = 5 mm, *t* = 10 mm, *d*_1_ = *d*_2_ = 0.4 mm, the charge energy utilization is 34.04%. The results of *d*_2_ parameter scan are shown in Fig. 8d. When *d*_2_ tends to 4–6 mm, the energy utilization tends stable. It was also found that a further reduction of the crucible thickness (*t*_1_ and *t*) would reduce the weakening of the magnetic flux and lead to a higher charge energy utilization. However, too thin wall could cause crucible burning, so a thin-walled crucible should correspond to the correct melting process parameters, e.g. cooling water flow, etc. When the crucible structure parameters are adjusted as *t*_1_ = 5 mm, *t* = 10 mm, *d*_1_ = 0.4 mm, *d*_2_ = 5 mm, the efficiency will increase from 27.1 to 38.3% for the charge in this research.

### Coil type

**Figure 9:**
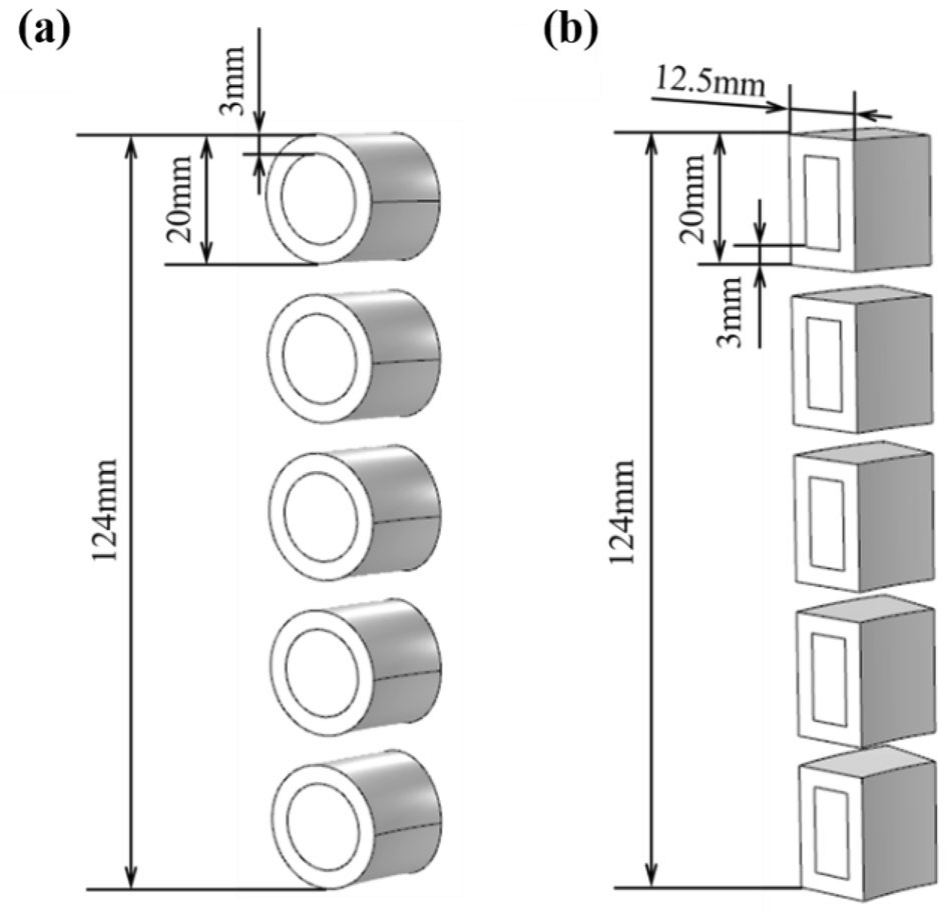
Coil with (**a**) circular cross section and (**b**) rectangular.

The initial coil cross-section was circular and we compared the effect of circular and rectangular coils on the magnetic intensity and charge energy utilization respectively in this subsection. Furthermore, the two types of coils have same cross-section area. The configurations were schematically set as shown in Fig. 9, which ensures the cross-section area per turn is about 160 mm^2^.

**Figure 10:**
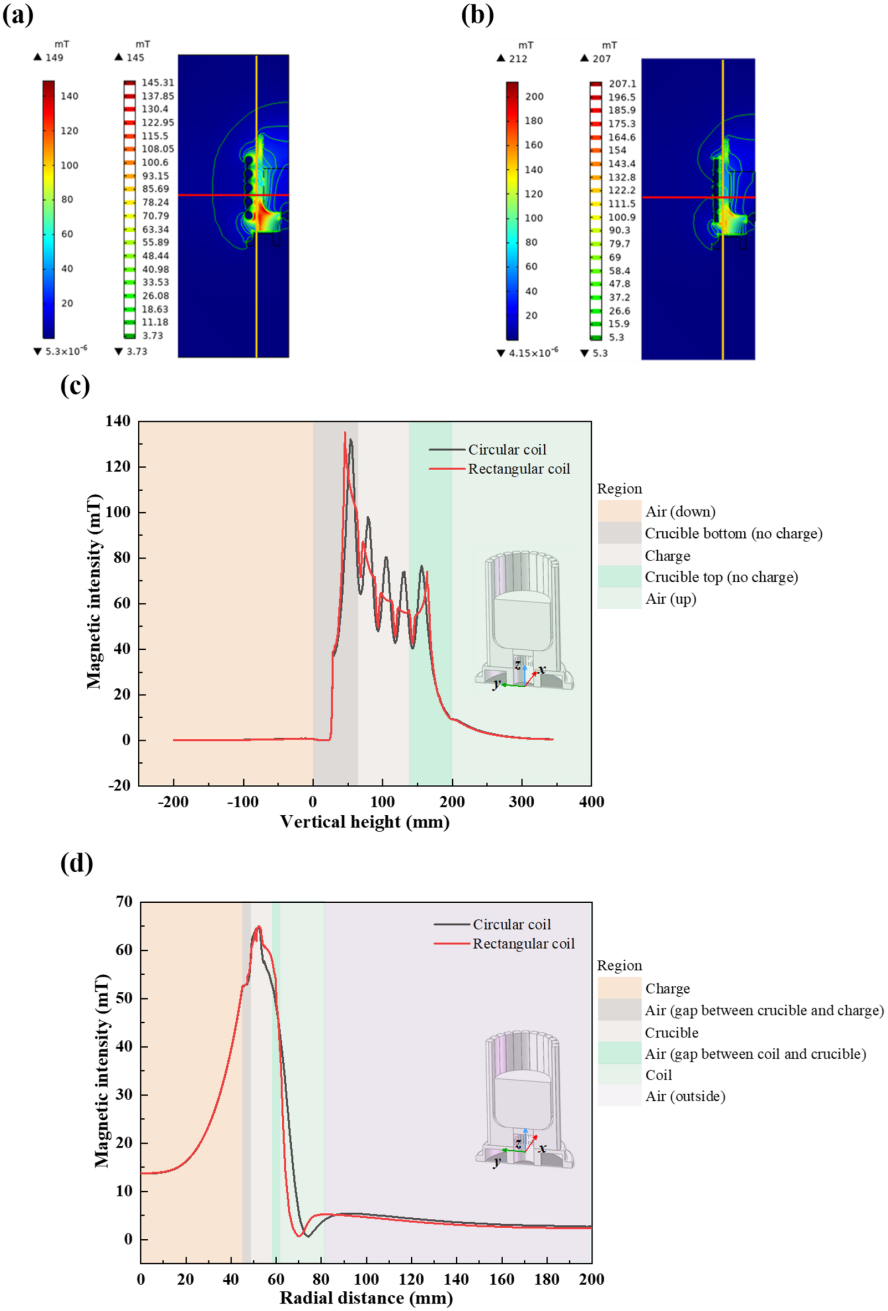
The magnetic field distribution between the slits for (**a**) circular and (**b**) rectangular coil; (**c**) vertical and (**d**) radial magnetic intensity of both coils.

The magnetic field distribution between the slits is shown in Fig. 10a,b for both, respectively. With the same induction power and frequency, both coils excite large magnetic intensity in the lower part of the crucible, and the maximum magnetic intensity is 149 mT for the circular coil and 212 mT for the rectangular. In addition, the magnetic leakage in the circular coil is more severe than in the rectangular, presumably related to the edge effect of the circular coil. In order to demonstrate the speculation, a comparison of the vertical and radial magnetic intensity was carried out. The location of the magnetic intensity data extraction is marked by yellow and red lines in Fig. 10a,b. The yellow line is located in the middle of the inner part of the coil and the outer part of the crucible; the red line is located in the middle of the second and third turns of the coil. The magnetic intensity of both is shown in Fig. 10c,d. The sine wave trend of the magnetic intensity exhibited by the circular coil confirms the same inference as before. In the vertical direction, the magnetic intensity of the circular coil fluctuates more sharply and is less uniform than the rectangular; in the radial direction, the magnetic intensity of both remains essentially the same. And the charge energy utilization has no significant change. A more homogeneous magnetic field will excite a more homogeneous electric field in terms of uniformity of heat distribution. In summary, the rectangular coil is better.

### Coil in series and parallel type

**Figure 11:**
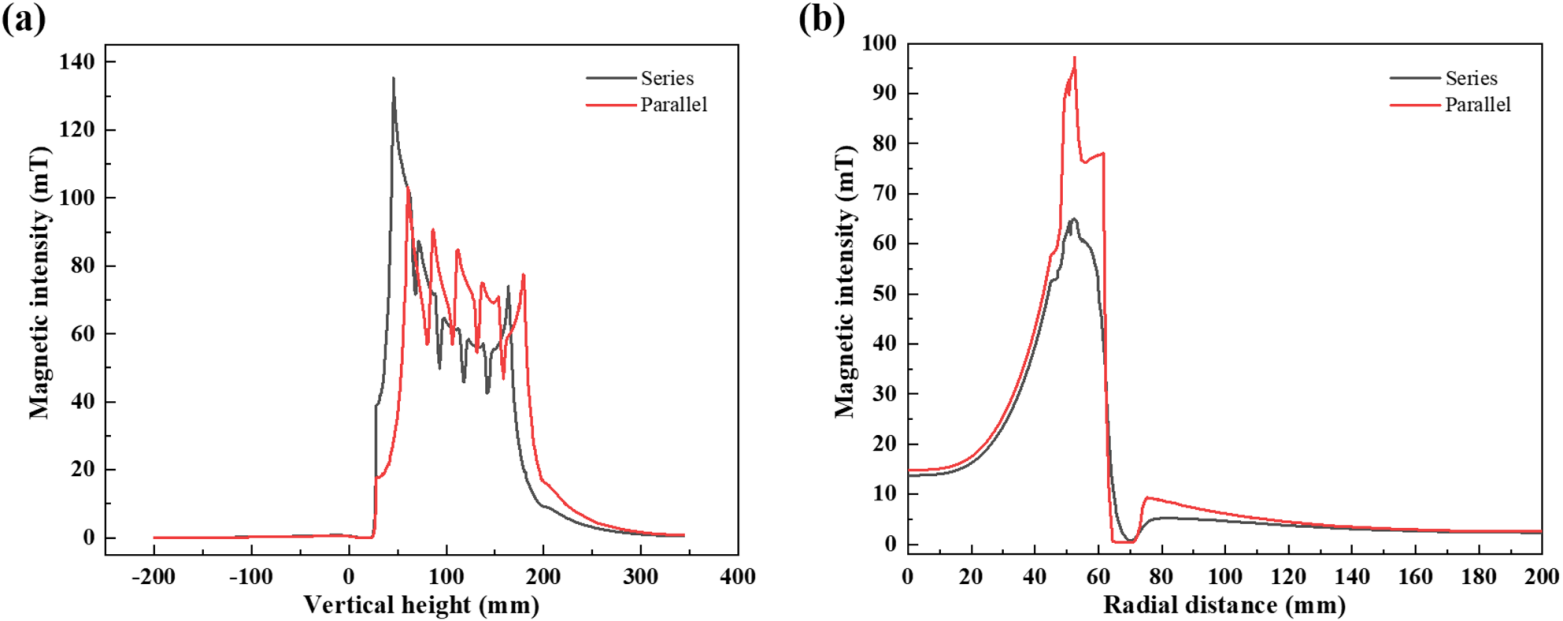
The magnetic intensity in (**a**) vertical and (**b**) radial direction of series and parallel type induction coil.

This subsection investigates the effect of coils connected in series or parallel on the magnetic field to study the charge energy utilization, all other parameters being fixed and coil type as rectangular. As in the previous position of yellow and red lines shown in Fig. 10a,b, the magnetic intensity excited by both is shown in Fig. 11a,b. In the vertical direction, the magnetic field is more homogeneous in parallel connection. It can be inferred that there exists path loss with series type. In the radial direction, the parallel excites a larger magnetic intensity on the charge. It may be caused by low electrical loss in the coil. The charge energy utilization increased from 37.35% (series) to 45.89% (parallel).

In addition, with the series type, if the current imbalance or sudden overload occurs in one turn, the other turns could be also affected at the same time. Furthermore, for increasing the coil turns number, the coil length increases, which increases the voltage between the coil terminals, which may induce arcing phenomenon. By connecting the coils in parallel, it is possible to reduce the voltage at both terminals and make the coil height control easier by manoeuvring power input mode. In our previous experiments, we found that the energy utilization and field uniformity on the charge was better when the coil height was the same as the melt meniscus height.

### Spacing between each turn

**Figure 12:**
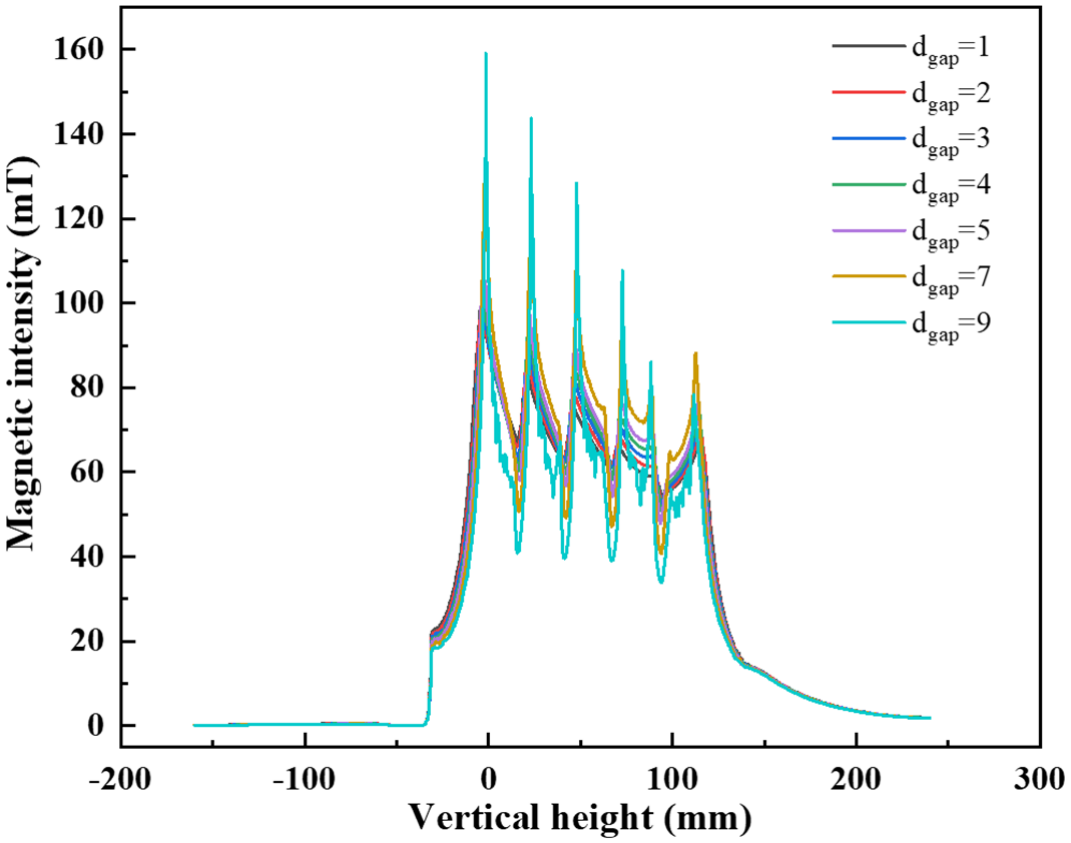
The vertical magnetic intensity varies with the spacing between each turn.

Coils can be classified as sparse or densely wound according to the size of their turn spacing. Keeping the coil total length, the effective cross-sectional area of each turn and other parameters unchanged, the vertical magnetic intensity varies with the turn spacing size as shown in Fig. 12. As the turn spacing increases, the magnetic leakage becomes more serious and the uniformity of the magnetic intensity is significantly reduced. Therefore, the reduction in turn spacing is beneficial for magnetic intensity homogeneity above the charge, which plays a positive role in soft contact and homogeneous heating. However, to prevent the breakdown of adjacent coil, the coil surface needs to be wrapped and coated with insulating paint. So, a certain space should be left for the insulation later between turns. Therefore, the smaller the gap between the coil turns, the better, provided that the insulation is maintained.

## Conclusions

The magnetic intensity and charge energy utilization with different cold crucible and coil structures in the ISM melting process were investigated by EM field numerical modelling. The conclusions are summarized as follows:

1. The crucible section number increase has a positive effect on charge energy utilization and magnetic intensity homogeneity. When the section number exceeds the threshold, further increases in the section number no longer significantly improve the magnetic intensity above the charge.
2. When there exist slits at the crucible bottom, the magnetic uniformity in the vertical direction of the charge increases, the magnetic flux is concentrated at the charge bottom and the current density converges at this position, playing an inductive heating effect. When the flat bottom crucible is changed to a curved shape, the EM force above the charge is increased.
3. Thinner wall and wide slits with the crucible bring a more positive impact on the charge energy efficiency. The new crucible structure has an 11.2% increase in energy utilization compared to the initial structure.
4. Rectangular cross section coil has a weaker edge effect and a more uniform excitation magnetic field than circular.
5. The parallel type coil has lower electrical loss compared to the series, the excitation magnetic intensity is higher, and the charge energy utilization is increased to 45.89%. In addition, the parallel coils also allow for better coil height control by manoeuvring whether power is input or not.
6. Reducing the coil turn spacing improves the charge magnetic uniformity and has an optimistic effect on the soft contact and uniform heating of the charge. Under the premise of ensuring safety, the turn spacing should be reduced as much as possible.

## Data Availability

The data that support the findings of this study are available from the corresponding author upon reasonable request.

## References

[1] Ranasinghe K, Guan K, Gardi A, Sabatini R (2019). Review of advanced low-emission technologies for sustainable aviation. Energy.

[2] González Palencia JC, Sakamaki T, Araki M, Shiga S (2015). Impact of powertrain electrification, vehicle size reduction and lightweight materials substitution on energy use, CO_2_ emissions and cost of a passenger light-duty vehicle fleet. Energy.

[3] Buliński P, Smolka J, Golak S, Przyłucki R, Palacz M, Siwiec G (2021). Analysis of Al–Zn alloy refining in an industrial induction skull melter with two crucible types. Int. J. Heat Mass Transf..

[4] Reed S (1995). Gamma titanium aluminide production using the induction skull melting (ISM) process. Met. Mater. Soc..

[5] Liu T, Dong Z, Zhao Y, Wang J, Chen T, Xie H (2012). Purification of metallurgical silicon through directional solidification in a large cold crucible. J. Cryst. Growth.

[6] Yanqing S, Jingjie G, Jun J, Guizhong L, Yuan L (2002). Composition control of a TiAl melt during the induction skull melting (ISM) process. J. Alloys Compd..

[7] Knyazev OA, Ptashkin AG, Stefanovsky SV, Zen’kovskaya MA, Yudintsev SV, Nikonov BS, Stefanovsky OL (2009). Inductive cold crucible melting and characterization of uranium and thorium bearing murataite ceramics. ICEM.

[8] Umbrasko A, Baake E, Nacke B, Jakovics A (2008). Numerical studies of the melting process in the induction furnace with cold crucible. COMPEL.

[9] Bojarevics, V., Pericleous, K., Harding, R. A., & Wickins, M. Cold crucible melting of reactive metals using combined DC and AC magnetic fields. In *EPM* 778–783 (2006).

[10] Jieren Y, Ruirun C, Yanqing S, Hongsheng D, Jingjie G, Hengzhi F (2018). Optimization of electromagnetic energy in cold crucible used for directional solidification of TiAl alloy. Energy.

[11] Buliński P, Smolka J, Golak S, Przyłucki R, Palacz M, Siwiec G, Lipart J, Bialecki R, Blacha L (2017). Numerical and experimental investigation of heat transfer process in electromagnetically driven flow within a vacuum induction furnace. Appl. Therm. Eng..

[12] Alcalá G, Rivero M, Cuevas S (2015). Effect of the magnetic field orientation on the damping of liquid metal free surface waves in the processing of materials. Appl. Therm. Eng..

[13] Chen R, Yang J, Ding H, Huang F, Su Y, Guo J, Fu H (2012). Effect of configuration on magnetic field in cold crucible using for continuous melting and directional solidification. Trans. Nonferr. Met. Soc..

[14] Fort J, Garnich M, Klymyshyn N (2005). Electromagnetic and thermal-flow modeling of a cold-wall crucible induction melter. Metall. Mater. Transf. B.

[15] Yang Y, Chen R, Guo J, Ding H, Su Y (2017). Experimental and numerical investigation on mass transfer induced by electromagnetic field in cold crucible used for directional solidification. Int. J. Heat Mass Transf..

[16] Jang J, Chiu Y (2007). Numerical and experimental thermal analysis for a metallic hollow cylinder subjected to step-wise electro-magnetic induction heating. Appl. Therm. Eng..

[17] Buliński P, Smolka J, Golak S, Przyłucki R, Palacz M, Siwiec G, Melka B, Blacha L (2018). Numerical modelling of multiphase flow and heat transfer within an induction skull melting furnace. Int. J. Heat Mass Transf..

[18] Yang J, Chen R, Ding H, Guo J, Han J, Fu H (2013). Thermal characteristics of induction heating in cold crucible used for directional solidification. Appl. Therm. Eng..

[19] Chen R, Ding H, Yang J, Guo J, Li X, Su Y, Fu H (2012). Directional solidification of Ti6Al4V ingots with an electromagnetic cold crucible by adjusting the meniscus. ISIJ Int..

[20] Spitans S, Jakovičs A, Baake E, Nacke B (2010). Numerical modelling of free surface dynamics of conductive melt in the induction crucible furnace. Magnetohydrodynamics.

[21] Buliński P, Smołka J, Golak S, Przyłucki R, Blacha L, Białecki R, Palacz M, Siwiec G (2015). Effect of turbulence modelling in numerical analysis of melting process in an induction furnace. Arch. Metall. Mater..

[22] Zhang C, Cao F, Zhang L, Jin Z, Cao G, Qiu Z, Shen H, Huang Y, Jiang S, Sun J (2023). Break the superheat temperature limitation of induction skull melting technology. Appl. Therm. Eng..

[23] Bojarevics V, Pericleous K (2004). Modelling induction skull melting design modifications. J. Mater. Sci.

[24] Harding, R. T. The use of combined DC and AC fields to increase superheat in an induction skull melting furnace. In *LMPC* 201–210 (2005).

[25] Buliński P, Smolka J, Siwiec G, Blacha L, Golak S, Przyłucki R, Palacz M, Melka B (2019). Numerical examination of the evaporation process within a vacuum induction furnace with a comparison to experimental results. Appl. Therm. Eng..

[26] Matsuzawa S, Yoshikawa G, Hirata K, Miyasaka F, Nakai Y, Tsuda M, Komemushi Y (2015). Coupled 3-D analysis employing FEM and particle method-experimental verification of cold crucible induction melting. IEEE Trans. Magn..

[27] Cho YW, Oh YJ, Yi KW, Chung SH, Shim JD (1996). Numerical analysis of molten metal shape in cold crucibles by 3D FEM. Model Simul. Mater. Sci. Eng..

[28] Umbrashko A, Baake E, Nacke B, Jakovics A (2005). Experimental investigations and numerical modelling of the melting process in the cold crucible. COMPEL.

[29] Lavers JD (2008). State of the art of numerical modeling for induction processes. COMPEL.

[30] Coppoli EHR, Mesquita RC, Silva RS (2012). Induction machines modeling with meshless methods. IEEE Trans. Magn..

[31] Nemkov, V., Goldstein, R., & Kreter, K.. Modelling and optimization of cold crucible furnaces for melting metals, In *Proceedings of the International Symposium Heating by Electromagnetic Sources* 21–24 (2013).

[32] Smalcerz A, Oleksiak B, Siwiec G (2015). The influence a crucible arrangement on the electrical efficiency of the cold crucible induction furnace. Arch. Metall. Mater..

[33] Dumont M, Ernst R, Garnier C, Hasan G, Petitpas P (2012). Innovative inductive cold crucible configurations with improved efficiency. J. Iron Steel Res. Int..

[34] Zhang C, Zhang L, Cao F, Jin Z, Cao G, Gao R, Qiu Z, Shen H, Huang Y, Jiang S, Sun J (2024). Fully coupled numerical analysis for induction skull melting technology applied for metallic glass component alloy. Int. J. Therm. Sci..

[35] Feng D, Luo H, Zou D, Zhang C (1994). Cold crucible induction suspension melting technology. J. Iron Steel Res..

[36] Ernst, R., Garnier, C., Petitpas, P., & Trassy, C. 2D and 3D numerical modelling of a cold crucible for optimizing of industrial processes. In *International Symposium on Heating by Electromagnetic Sources* (2007).

[37] Dumont, M., Ernst, R., Garnier, C., Petitpas, P. Dumont, M., Ernst, R., Garnier, C. et al. Recent progresses in optimizing inductive cold crucible processes. In *EPM* 19–23 (2009).

[38] Chen R, Ding H, Bi W, Guo J, Jia J, Fu H (2005). Electromagnetic cold crucible technology and its applications. Rare Met. Mater. Eng..

